# Fiber Structure-Property Relationships: A Disulfide-Crosslinked Self-Crimping Polyamide

**DOI:** 10.6028/jres.065A.052

**Published:** 1961-12-01

**Authors:** Stephen D. Bruck

## Abstract

A structural modification of nylon-6 fiber (polycaprolactam) was achieved by the introduction of a high density of intermolecular disulfide crosslinkages. The crosslinking process leads to an unexpected three-dimensional crimping in the dry and wet states (similar to wool), and to the formation of helical coils if swelling is carried out in a solvent capable of destroying the crystallites remaining after crosslinking. This phenomenon has not been observed previously in round cross-section synthetic homofibers. A possible explanation is advanced which attributes this crimping and coiling tendency to differential swelling caused by the varying crosslinking density across the fiber axis and to structural asymmetry resulting from the crosslinking process.

## 1. Introduction

Although the use of crosslinking reactions has markedly changed the properties of rubber, very little work has been published on the chemical intermolecular crosslinking of oriented, semicrystalline structures, such as synthetic fibers. Some work which has appeared in the literature deals with crosslinking reactions on dissolved polymer systems, but such preformed networks cannot be readily spun and drawn into fibers. It seems reasonable to expect, however, that at least some of the numerous known organic reactions could be utilized to bring about structural changes in synthetic homofibers which could markedly affect their properties.

This paper deals with the structural modification of nylon-6 fiber (polycaprolactam) as the result of a four-step disulfide crosslinking reaction. It will be shown that under suitable conditions this process leads to unexpected three-dimensional crimping in the dry and wet states (similar to wool), and to the formation of helical coils if swelling is carried out in a solvent capable of destroying the crystallites remaining after crosslinking. This phenomenon has not been observed previously in round cross-section synthetic homofibers, such as nylon-6.

In general, crimping in synthetic fibers can be induced either by mechanical means or by the preparation of heterofibers in which two polymers of different shrinkage characteristics are spun side-by-side from the same spinneret giving fibers with bilateral structures [1]^1^. Among the natural fibers, wool has been found to have a bilateral, heterostructure composed of the orthocortex and the paracortex [2]. Crimped rayon staple is being made by the spinning of asymmetrical cross-section viscose into a specific low-acid coagulating bath and subsequently stretching the filament bundle in a hot bath [3].

## 2. Introduction of Intermolecular Disulfide Crosslinks

The existence of disulfide crosslinkages in wool fibers and other proteins is well known. Many of the unique properties of these systems have been attributed to these crosslinkages. The relative ease with which the sulfhydril groups may be oxidized to the disulfide linkages offers a promising approach to the study of chemical reactions which could lead to similar crosslinking of synthetic fibers. Polycaprolactams appear to possess the necessary requirements by virtue of their amide groups. Instead of using dissolved polycaprolactams, the use of already spun and drawn fibers offers a greater challenge inasmuch as such systems have a “build-in” crystallinity and orientation which should influence the final properties. Thus, for the present study nylon-6 fiber was chosen.

Early work by Cairns and coworkers [4, 5] described the preparation and some properties of *N*-methylol, *N*-alkoxymethyl, and *N*-alkylthiomethyl polyamides. Although these reactions were carried out while the polymers were in the dissolved state, they are of interest as potential precursors for crosslinking. It seemed desirable to investigate techniques aimed at the further development and application of some of these reactions on solid structures with the objective of introducing a large number of disulfide crosslinks into nylon-6 fiber.

The following chemical reactions are involved (for details of procedure, refer to Experimental section):

**Figure:**
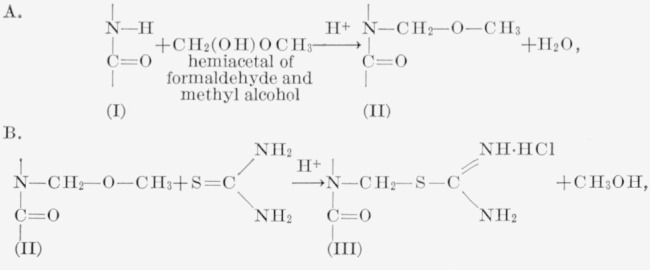

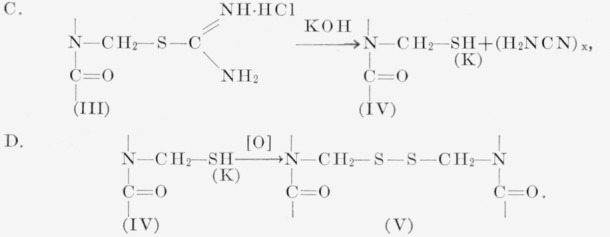


Step A involves the reaction of the amide hydrogen with the hemiacetal of formaldehyde and methyl-alcohol in the presence of strong acids, producing an *N*-methoxy-methylated polycaprolactam (II). The structure of this product has already been established [4] from the facts (a) *N*-methoxy-methylated polyamides produce formaldehyde in good agreement with the expected quantities predicted from methoxyl analyses, (b) polyamides prepared from *N*,*N*′-alkylated diamines do not react with the hemiacetal of formaldehyde and methyl alcohol, and (c) infrared absorption spectroscopy indicates a substantial decrease in the intensity of certain −NH absorption bands in the *N*-methoxymethylated polyamides.

In the present work, the *N*-methoxymethylation of solid nylon-6 fiber resulted in the formation of 3 to 4 percent methoxyl (depending on the experimental conditions used) which corresponds to an amide substitution in the polymer of approximately 11 to 15 percent. X-ray diffraction patterns of the *N*-methoxymethylated and unmodified fibers show no change of crystallinity, indicating that most likely only the amorphous regions are penetrated by the reagents. Since nylon-6 fiber is about 50 percent crystalline, 22 to 30 percent of the amide groups in the amorphous regions of the polymer were methoxymethylated.

Steps B and C involve the reaction of the methoxymethylated fiber with thiourea in the presence of strong acids and subsequent treatment with alkali to yield the sulfhydril product (IV). These reactions are analogous to the preparation of methylmercaptans from thiourea and alkyl halides (6):

**Figure:**
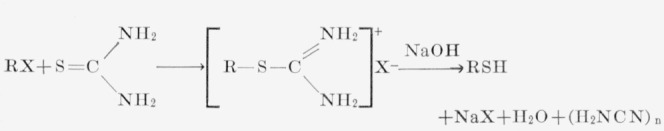


To determine the effect of the reaction medium on the extent of reactions A, B, and C, experiments were carried out both in water and in methyl alcohol, respectively, since the latter is a plasticizer for *N*-methoxymethylated polycaprolactams. In case of the experiments in water, methoxymethylated nylon-6 fibers were treated with thiourea and hydrochloric acid under various conditions, and then reacted with potassium hydroxide with concurrent air oxidation. Thus, the final structure contained both sulfhydril groups and disulfide crosslinks. Table 1 (samples 1 to 4) summarizes the pertinent analytical data. The percent of sulfur represents the combined quantities of sulfhydril groups and disulfide crosslinkages. The sulfur content of the samples varied from about one to two percent, depending on the conditions used.

The extent of crosslinking could also be estimated by equilibrium volume swelling measurements developed by Bruck [7] using a solvent (such as *m*-cresol) that could dissolve the uncrosslinked fiber. The symbol *q_m_* represents the ratio of the volumes of the swollen to the unswollen structures, *V/V*_o_ at equilibrium [8]. The observed *q_m_* values (table 1) are rather large indicating relatively few crosslinkages; this could be expected from the sulfur analyses, and from the mild air-oxidation treatment.

Although only a moderate number of crosslinks were present, cross sections of these fibers indicated that the intermolecular disulfide crosslinkages are not confined to the surface of the fiber but extend throughout the entire structure [9]. It is quite possible, however, that a larger number of crosslinks are located nearer to the surface than towards the center of the filaments. The significance of this will be discussed in a later section of this paper.

In another series of experiments methyl alcohol was used as the reaction medium. The plasticizing effect of this solvent on *N*-methoxymethylated polycaprolactams facilitates the opening of the amorphous regions with some decrease in the crystallinity of the fiber. The experiments were carried out at room temperature since hot methyl alcohol partially dissolves the methoxymethylated fiber. Table 1 (samples 5 to 7) summarizes the results. The total quantities of sulfur (representing sulfhydril groups as well as disulfide crosslinkages) varied from 2.7 to 3.5 percent depending on the conditions used, a substantial increase over the reactions that bad been carried out in water. Increased crosslinking is reflected by the decreased *q_m_* values.

Birefringence measurements which were carried out on unmodified, and on methoxymethylated nylon-6 fibers (using a slot compensator and sodium D light) disclosed only small variations along a given fiber axis of approximately 2″ length. Birefringence = (*n*_1_−*n_2_*) =*R/t*, where *n*_1_ and *n*_2_ are the refractive indices along and across the fiber axis, respectively, *R*=retardation in millimicrons (measured with the compensator), and *t*=thickness of the fiber in millimicrons. The birefringence values of these samples varied between +0.055 and +0.060. Furthermore, X-ray diffraction patterns showed no appreciable changes in the total crystallinity of these samples, a good indication that the *N*-methoxymethylation reaction is confined largely to the amorphous areas of the fiber [10]. Birefringence measurements on the disulfide-crosslinked structures that had been treated in water showed only small fluctuations along a given fiber axis, unlike those samples that bad been treated in methanol. These latter samples showed birefringence values that ranged from +0.028 to +0.049 for five measurements along approximately 2″ length specimens. The reason for this may be the partial disruption of the crystallites due to the plasticizing effect of methyl alcohol and the structural strain caused by the intermolecular crosslinks.

In order to determine the effect of oxidizing agents other than air on the formation of disulfide crosslinks from sulfhydril groups, hydrogen peroxide and iodine (both well-known oxidizing agents for sulfhydril groups) were investigated. The samples described in table 1 were further subjected to oxidation by H_2_O_2_. When dilute solutions of the peroxide were used for 30 min, additional crosslinkages appeared in the polymers over those already introduced by air oxidation. This was evidenced by decreased *q_m_* values as summarized in table 2. On the other hand, more concentrated solutions of H_2_O_2_ (or I_2_) caused injury to the fibers which is not surprising considering the general sensitivity of nylons to prolonged exposure to strong oxidizing agents.

## 3. Crimping of the Disulfide-Crosslinked Fibers

**Figure 1:**
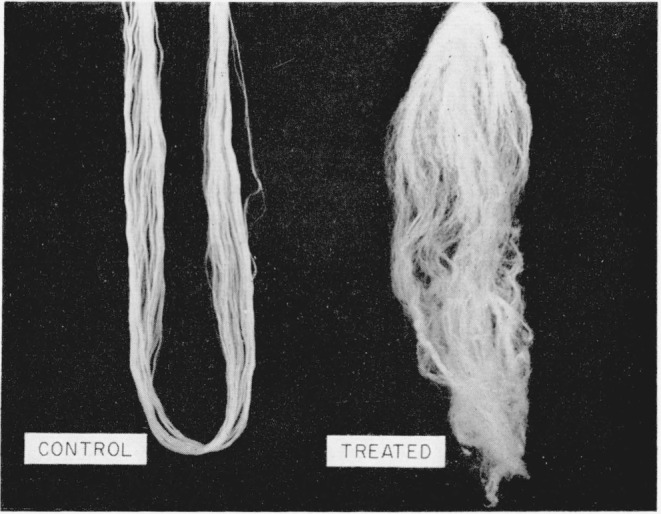
Unmodified and disulfide-crosslinked nylon-6 fibers.

**Figure 2:**
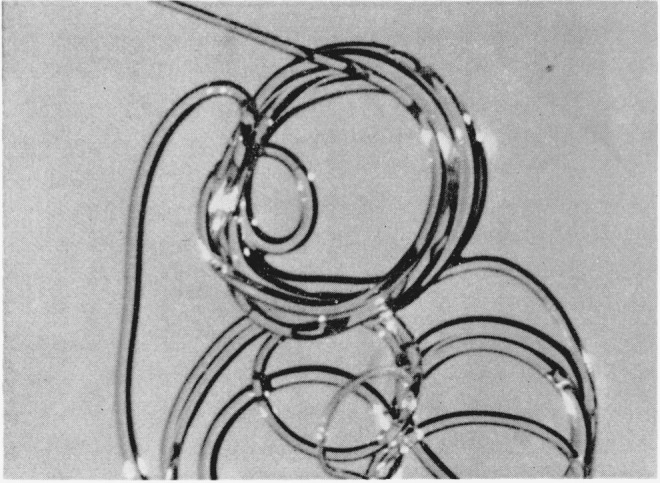
Helical coiling of disulfide- crosslinked nylon-6 fiber.

**Figure 3:**
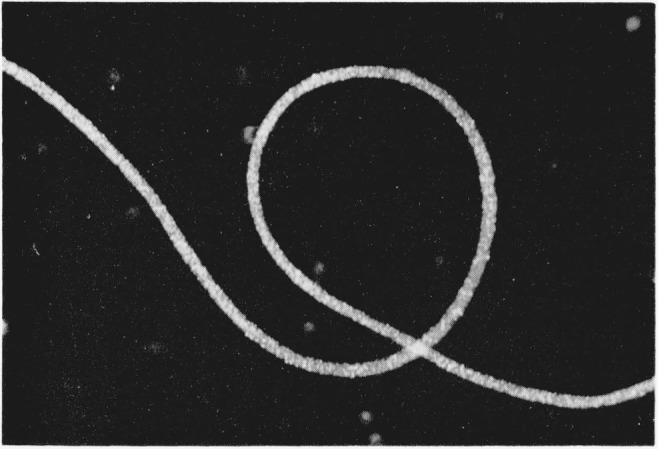
A single helical coil of disulfide- crosslinked nylon-6 fiber.

**Figure 4:**
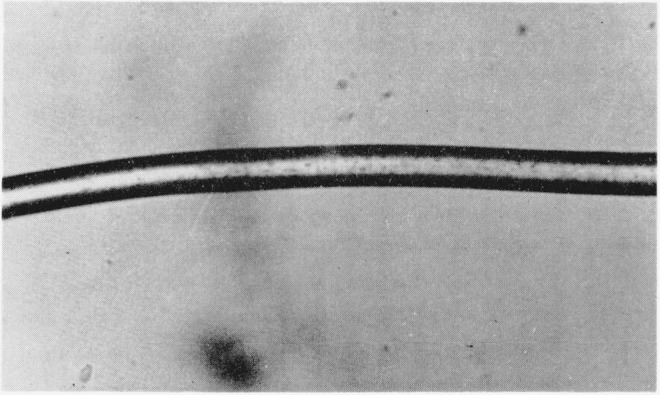
Photomicrograph of unswollen disulfide- crosslinked nylon-6 fiber (under restraint).

**Figure 5:**
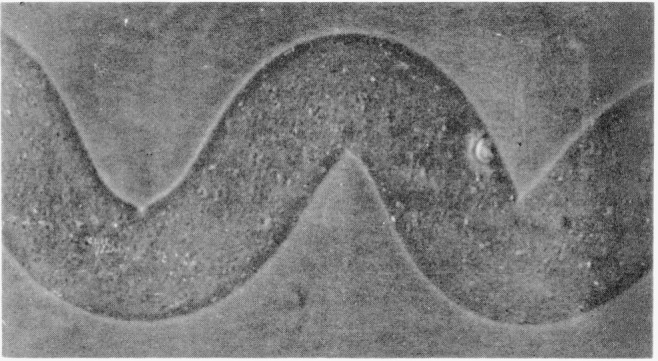
Phase-photomicrograph of swollen (m-cresol) disulfide- crosslinked nylon-6 fiber (under restraint).

The disulfide-crosslinked nylon-6 fibers exhibited unexpected three-dimensional wavy crimping of uneven distribution and dimensions, both in the wet and dry states, similar to wool. This crimping was especially pronounced in those fibers that had been treated in the presence of methyl alcohol and crosslinked by either air oxidation or by dilute solutions of H_2_O_2_ for 30 min. Figure 1 is a photograph of these fibers. Much reduced, but still noticeable crimping was also exhibited by those samples that had been crosslinked in the presence of water instead of methyl alcohol. On the other hand, helical coiling was produced by either series of fibers when they were treated with *m*-cresol which destroyed the remaining crystallites, thus removing the opposing force to coiling. Figures 2 and 3 are photographs of the helical coils produced after swelling in *m*-cresol. Corresponding phase-photomicrographs are illustrated by figures 4 and 5 before and after swelling, respectively (fibers under restraint), under identical magnifications.

As noted earlier, the formation of three-dimensional crimping and helical coiling has not been previously observed with round cross-section (symmetrical) synthetic homofibers, such as nylon-6. Thus, unlike wool, the homofiber has no “built-in” bilateral asymmetry. However, the observed crimping and coiling is undoubtedly due to some structural strain and asymmetry induced by the crosslinking process. Although the exact nature of this phenomenon is not clear at this time, a possible explanation will be attempted.

**Figure 6:**
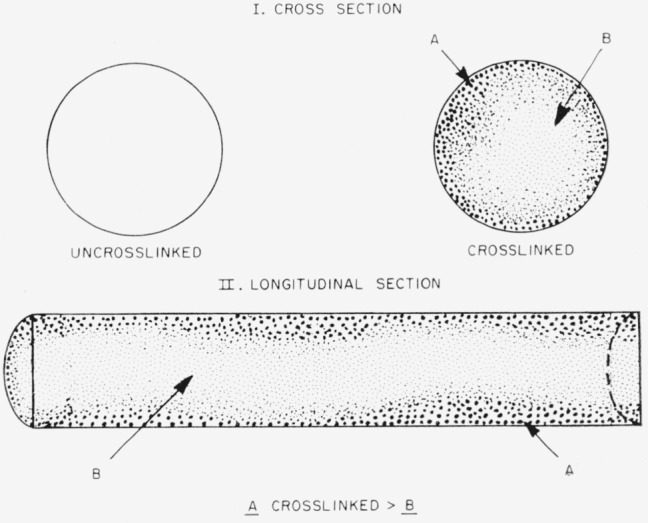
Schematic illustrations of the crosslinked fibers.

It was stated previously that the final oxidation step of sulfhydril groups to disulfide crosslinks was brought about by either air oxidation, or by relatively brief treatment (30 min) with dilute solutions of hydrogen peroxide. It is reasonable to assume that during this treatment those sylfhydril groups that are located nearer to the surface of the fiber will be oxidized to a greater extent than those situated towards the center. Furthermore the onset of the oxidation reaction will be accelerated at points along the fiber axis that are easily penetrated. As discussed already, step A in the chemical reaction series was carried out in the presence of hydrochloric acid which could cause structural nonuniformity in the fiber with the result that crosslinking agents will be able to penetrate certain regions more easily than others. Thus asymmetry should result within the round fibers by virtue of the varying crosslinking density across the fiber cross section. Although the fiber is crosslinked throughout, it might have an asymmetrical outer portion with a higher degree of crosslinking, and an inside asymmetrical portion with fewer crosslinks. A schematic illustration of this situation is given in figure 6. During swelling in water, the inner, less crosslinked parts will swell more than the outer more crosslinked portions. Water, which is a poor swelling agent, penetrates primarily the amorphous portions of the fiber and hence a counterforce is maintained as long as the crystallites remain intact. The relatively small differentia] swelling in water therefore is insufficient to overcome the masking effect of the crystallites which remain dominant, especially in view of the fact that the original fiber was approximately 50 percent crystalline. Under such conditions only crimping but no coiling is observed. During swelling in *m*-cresol, which completely destroys the crystallites, the differential swelling caused by the differential crosslinking density is no longer masked by the crystallites and this results in helical coiling of the fiber.

The fact that the fibers are crosslinked throughout and not just near the surface is of utmost importance. Fibers in which a different type of crosslinking was restricted to near the surface produced a hole in the middle of the fiber when cross sections were treated with swelling agents capable of dissolving the uncrosslinked structure [9]. In such fibers there is no differential swelling process and hence no crimping and coiling are observed.

The critical importance of the differential swelling of the disulfide crosslinked fiber as the result of varying crosslinking density may also be demonstrated as follows. When those crosslinked fibers in which the final oxidation step was carried out with air or with dilute hydrogen peroxide solutions for 30 min were later subjected to additional, more prolonged oxidation by dilute H_2_O_2_, they exhibited greatly diminished crimping and coiling tendencies. This is most likely due to the more uniform distribution of crosslinks throughout the fiber as the result of better penetration by the oxidizing agent.

It may be desirable to stabilize the system to prevent any possible gradual slow oxidation throughout the fiber by air which could eventually negate the crimping and coiling effects. One possible way to avoid this is to treat the crosslinked and partially oxidized fiber with AgNO_3_, according to the following equation [11]:

−SH+Ag+→−SAgI II

Product II is thus blocked from further oxidation and the fiber structure is “frozen” in the desired state.

Although in this work *m*-cresol was used to effect the complete destruction of the crystallites and hence produce maximum helical coiling, the judicious use of other swelling agents alone or in combination could result in various degrees of helical coiling depending on the extent of crystallinity of the fiber.

## 4. Experimental Procedure

All work was carried out with 7.8 Tex (60 denier)/32 filament nylon-6 fiber, polymer molecular weight 14,000 (end group analysis).

### 4.1. *N*-methoxymethylation[13]: Step A

A small skein of nylon-6 fiber, weighing approximately 0.1 to 0.2 g was scoured for 30 min in water containing 1 to 2 percent Na_3_PO_4_, washed in distilled water, and dried.

A solution was prepared containing 500 g paraformaldehyde and 500 g (625 ml) methyl alcohol by heating the solution to 60 °C and adding 3 to 4 pellets of KOH. The solution was stirred at this temperature until all paraformaldehyde dissolved (approx. 15 min) and then it was allowed to cool to room temperature. The *p*H of the solution was then adjusted to 0.6 to 0.7 with anhydrous oxalic acid (approx. 40 g). The skein was soaked in this solution for 12 hr at room temperature after which it was removed from the bath and heated at 120 ° C in a closed oven for 1 min, rinsed in methylalcohol followed by water, and dried. Methoxyl analysis [12]: 3.4%.

### 4.2. Introduction of Sulfhydril Group and Oxidation to Disulfide Crosslinks: Steps B, C, and D

#### Exp. No. 1

The *N*-methoxymethylated nylon-6 c containing 38.0 g thiourea (0.5 mole), 700 ml distilled water, and 35 ml cone. HC1 (0.42 mole) at room temperature for 12 hr. After this period 28.0 g (0.5 mole) KOH was added in 100 ml distilled water and the fiber was permitted to soak at room temperature for 12 hr with concurrent air oxidation from a porous-disk bubbler. Next, the fiber was removed from the solution, thoroughly washed with distilled water, and dried. *S*=2.1%.

#### Exp. No. 2

Same as above, except that fiber was soaked for 1 hr in the thiourea, H_2_O, and HC1 mixture and for 1 hr in KOH. *S*=1.0%.

#### Exp. No. 3

The *N*-nethoxymethylated nylon-6 fiber was soaked in a solution containing 38.0 g thiourea (0.5 mole) and 700 ml distilled water for 12 hr at room temperature. Next, 35 ml conc. HC1 (0.42 mole) was added and the fiber soaked for 1 hr at room temperature. After this period 28.0 g (0.5 mole) KOH was added in 100 ml distilled water and the fiber was permitted to soak at room temperature for 1 hr with concurrent air oxidation from a porous-disk bubbler. The fiber was then washed with distilled water and dried. *S*=1.3%.

#### Exp. No. 4

The *N*-methoxymethylated nylon-6 fiber was soaked in a solution containing 38.0 g thiourea (0.5 mole) and 700 ml distilled water for 2 hr at 60 °C. The solution was cooled to room temperature and 28.0 g (0.5 mole) KOH was added in 100 ml distilled water; the fiber was soaked in this solution for 1 hr at room temperature with concurrent air oxidation from a porous-disk bubbler. The fiber was then washed with distilled water and dried. *S*=1.0%.

#### Exp. No. 5

Same as Exp. No. 1, except that methyl alcohol was used instead of water as the reaction medium. *S*=2.9%.

#### Exp. No. 6

Same as Exp. No. 2, except that methyl alcohol was used instead of water as the reaction medium. *S*=3.5%.

#### Exp. No. 7

Same as Exp. No. 3, except that methyl alcohol was used instead of water as the reaction medium. *S*=2.7%.

##### Oxidation of Sulfhydril Groups to Disulfide Linkages with H_2_O_2_

The skeins from Exp. 1 to 7, respectively, were soaked at room temperature in a solution consisting of 250 ml distilled water, 2 pellets of KOH, and 10 ml of 3 percent H_2_O_2_ for 30 min to 4 hr, depending on the particular experiment.

## Figures and Tables

**Table 1:** Analytical data on structurally modified nylon-6 fibers^1^

No.^2^	Total S^3^	Reaction medium	Amide substitution	Swelling ratio, q*_m_*^4^	Comments	
					Dry or wet	*m*-cresol
						
	%		%			
1	2.1	Water	7.4	11.7	Very few crimps.	Helix
2	1.0	do	3.6	14.4	do	Do.
3	1.3	do	4.8	6.9	do	Do.
4	1.0	do	3.6	8.5	do	Do.
5	2.9	Meth anol.	10.3	6.7	Highly crimped.	Helix
6	3.5	do	12.4	4.3	do	Do.
7	2.7	do	9.5	4.3	do	Do.

1 *N*-methoxymethylated nylon-6: 3.4% methoxyl groups.

2 For reaction conditions refer to Experimental section.

3 Total sulfur includes both sulfhydril groups and disulfide crosslinkages.

4 Measured photomicrographically after 24 hr air-oxidation *q_m_=V/V*_o_ where *V*=volume of network at equilibrium swelling, *V*_o_=volume of network before swelling. Swelling agent: *m*-cresol.

**Table 2:** Equilibrium volume swelling ratios^1^ of samples oxidized by air and hydrogen peroxide

Sample No.	Oxidized by air–24 hr	Oxidized by H_2_O_2_–30 min	Oxidized by H_2_O_2_–4 hr
			
	*q_m_*	*q_m_*	*q_m_*
5	6.7	5.9	5.5
6	4.3	3.9	3.5
7	4.3	4.1	3.9

1 Measured photomicrographically, *q_m_=V/V*_0_, where *V*=volume of network at equilibrium swelling, *V*_0_=volume of network before swelling. Swelling agent: *m*-cresol.

## Footnotes

1 Figures in brackets indicate the literature references at the end of this paper.
